# Traumatic rupture of anterolateral papillary muscle in a patient with congenital deficiency of left hemi-pericardium: case report

**DOI:** 10.1093/jscr/rjad274

**Published:** 2023-06-19

**Authors:** K A Adomako, M N Tamatey, G Offei-Larbi, L A K Sereboe, I Okyere

**Affiliations:** Department of Surgery, Cardiothoracic Unit,37 Military Hospital, Accra, Ghana; Department of Surgery, School of Medicine, University of Health and Allied Sciences, Ho, Ghana; Department of Surgery, Cardiothoracic Unit, University of Ghana Medical School, Legon, Accra, Ghana; National Cardiothoracic Centre, Korle-Bu Teaching Hospital, Accra, Ghana; Department of Surgery, School of Medicine and Dentistry, College of Health Sciences, Kwame Nkrumah University of Science and Technology, Kumasi, Ghana

## Abstract

Congenital absence or deficiency of the pericardium or hemi-pericardium is uncommon. When it happens, the protection given to the heart is greatly reduced, and blunt or penetrating trauma to the chest is transmitted directly to the heart. Valvular injuries from these traumas are however rare with a case of tricuspid valve rupture reported. Traumatic papillary muscle rupture in all patients group is also rare. Co-existing rupture of papillary muscle in patients with congenital absence of the pericardium is extremely rare. We report the rare case of a patient with deficient left hemi-pericardium who had a traumatic rupture of the anterolateral papillary muscle from a road traffic accident.

## INTRODUCTION

Cardiac injury secondary to blunt chest trauma is quite common and may affect any cardiac structure depending on the force of impact [1]. Traumatic injury to the mitral valve is, however, rare, while traumatic papillary muscle rupture is very rare with 24 cases of papillary muscle rupture in 546 autopsy cases of non-penetrating trauma to the chest [2]. As of 2001, only 25 cases of surgically corrected post-traumatic mitral regurgitation were reported of which only 8 resulted from the rupture of the anterolateral papillary muscle [3]. Patients with congenital absence or deficiency of the pericardium are uncommon with only about 200 reported cases. Coexisting congenital pericardial defect with traumatic valve injury is extremely rare, with one reported case of traumatic tricuspid valve rupture reported by Giovanni et al in 2009 [2]. We report a case of traumatic rupture of the anterolateral papillary muscle in a patient with congenital deficiency of the left hemi-pericardium.

## CASE REPORT

A 44-year-old male presented with a 3-week history of congestive heart failure, NYHA II, which rapidly deteriorated to NYHA IV. There was associated constrictive central chest pain and palpitations but no syncope or fever.

He had two previous road traffic accidents both on a motor bicycle. The first was 7 months prior to presentation, when he had a blunt chest injury and a fracture of his left leg. The second was a month prior to presentation, when he sustained a dislocation to his right wrist as well as a fracture to his right radius.

**Figure 1:**
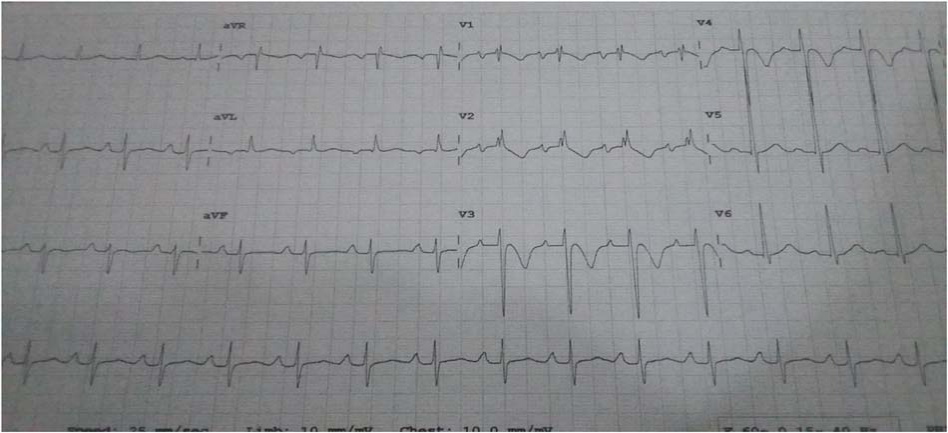
ECG of the patient.

**Figure 2:**
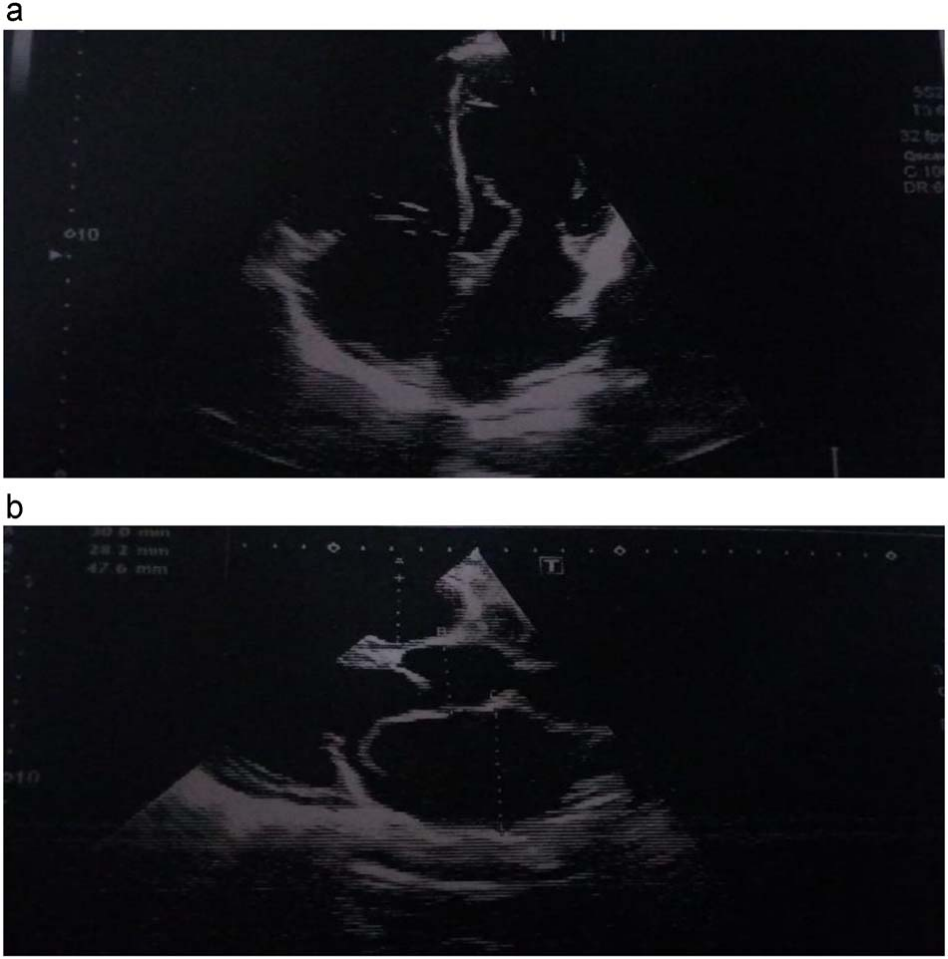
(**a**, **b**) Echocardiographic picture showing the avulsed papillary muscle.

**Figure 3:**
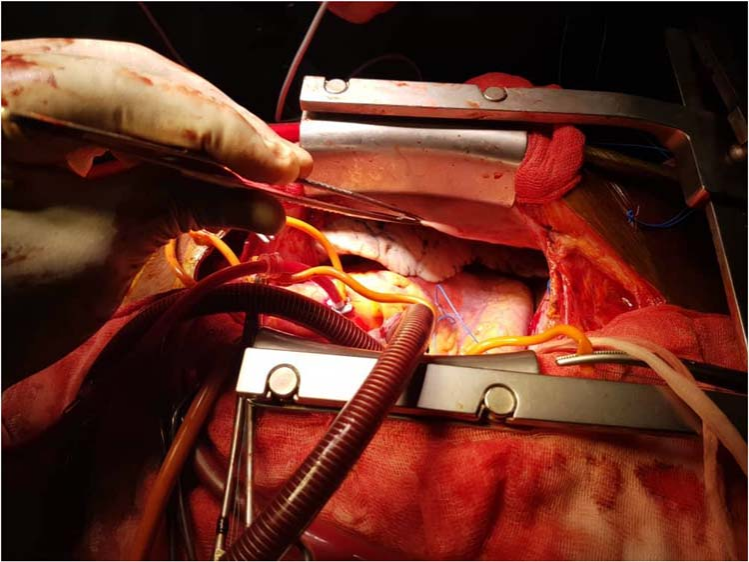
(**a**, **b**) Intraoperative pictures showing the deficient left hemi-pericardium.

**Figure 4:**
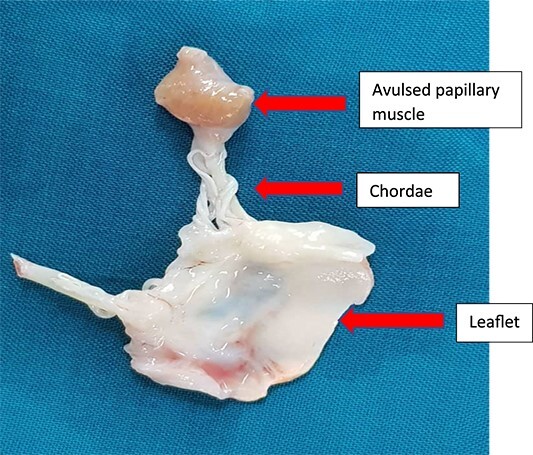
Excised mitral valve leaflet with the avulsed papillary muscle.

He was dyspneic, not pale and afebrile. He had bilateral pitting pedal edema up to the knees. Pulse rate was 102 regular non-collapsing, and blood pressure was 96/75 mmHg. The precordium was active, with the apex beat located in the sixth left inter-coastal space anterior axillary line. The first and second heart sounds were present, with an apical pan systolic murmur, which radiated to the left axilla. The respiratory rate was 20 cycles/min, with bi-basal crepitations on auscultation. The abdomen and other systems were normal. The Chest X-ray showed cardiomegaly with pulmonary plethora. The echocardiography (ECG) (Fig. 1) showed sinus tachycardia with inverted T-waves in the anterolateral leads. The ECG (Fig. 2a and b) showed normal global and segmental contractility with an ejection fraction of 75%. There was moderate dilation of the left atrium, with severe mitral regurgitation secondary to anterior mitral valve prolapse caused by rupture of the anterolateral papillary muscle. The intraoperative findings (Figs 3a, b and 4) were a deficient left hemi-pericardium, and rupture of the anterolateral papillary muscle. He had mitral valve replacement with a mechanical prosthetic valve. The postoperative period was uneventful, and the patient was discharged from the hospital after 9 days.

## DISCUSSION

Cardiac injury secondary to blunt thoracic trauma is quite common (about 20%); however, associated valvular injury is rare with about 82 reported cases worldwide [1, 2]. In valvular lesions, the aortic valve is most commonly affected, followed by the mitral and tricuspid valves in that manner. Isolated mitral valve injury is rare and usually involves rupture or avulsion of papillary muscle or chordae or tears of the leaflets [3].

Traumatic injury to the mitral valve is rare, while traumatic papillary muscle rupture (Fig. 4) is rare, with only 24 cases reported in 546 autopsy cases of non-penetrating trauma to the chest [1].

Patients with congenital absence or deficiency of the pericardium (Fig 3a and b) are uncommon with only about 200 reported cases. Coexisting congenital pericardial defect with traumatic valve injury (Figs 3a, b and 4) is extremely rare, with only one reported case of traumatic tricuspid valve rupture in a patient with a pericardial defect in 2009 [4] but none has been reported of the rupture of the anterior papillary muscle of the mitral valve coexisting with congenital absence of the pericardium.

Traumatic papillary muscle rupture usually affects males between the ages of 30 and 60 years and occurs equally in all races. The heart is sandwiched between the sternum and the vertebrae, thus rendering it to any anteroposterior compression of the thoracic cage. Traumatic rupture of the mitral valve or its attachments occurs in diastole when the heart is filled and is violently compressed with pressures as high as 320 mmHg [1]. This alone or with any left ventricular outflow tract obstruction causes a sudden rise in intra-ventricular pressure, which ruptures the chordae, papillary muscles or valvular tissues [2].

The clinical presentation depends on the severity of the injury and ranges from vague chest pains to features of florid congestive cardiac failure and maybe in any class of the NYHA. They will usually present with a mitral regurgitant murmur, especially when hypovolemia has been corrected.

Investigations include a chest X-ray which may show cardiomegaly with plethoric lung fields. An electrocardiogram may show sinus tachycardia. The gold standard for diagnosis is transesophageal echocardiography which has higher resolution than trans-thoracic and can confirm the rupture of a papillary muscle.

The treatment involves mitral valve replacement when there is an avulsion or rupture of a papillary muscle from the ventricular wall, or mitral valve ring annuloplasty where applicable [5].

## CONCLUSION

Congenital deficiency of the left hemi-pericardium is rare. This defect puts the heart at risk of injuries from chest trauma. One of such injuries is the rupture of the anterolateral papillary muscle of the mitral valve. We present such a case, which was managed with mitral valve replacement, with a good outcome.

## CONFLICT OF INTEREST STATEMENT

None declared.

## FUNDING

None.
